# Benefits, Facilitators, and Recommendations for Digital Health Academic-Industry Collaboration: A Mini Review

**DOI:** 10.3389/fdgth.2021.616278

**Published:** 2021-04-15

**Authors:** Kelsey L. Ford, Jennifer D. Portz, Shuo Zhou, Starlynne Gornail, Susan L. Moore, Xuhong Zhang, Sheana Bull

**Affiliations:** ^1^Colorado School of Public Health, University of Colorado, Anschutz Medical Campus, Aurora, CO, United States; ^2^School of Medicine, University of Colorado, Anschutz Medical Campus, Aurora, CO, United States

**Keywords:** academic industry collaboration, digital health, innovation, partnership, intersector collaboration

## Abstract

Digital health remains a growing and challenging niche in public health practice. Academic-industry collaboration (AIC) offers a mechanism to bring disparate sectors together to alleviate digital health challenges of engagement, reach, sustainability, dissemination, evaluation, and equity. Despite the ongoing endorsements for AIC in digital health, limited understanding exists of successful AIC exists. Most published research highlights the barriers of collaboration rather than efficacy, leaving collaborators asking: What are the benefits and facilitators of AIC and do they apply in digital health? As an initial effort to fill the gap in the literature, the purpose of this mini review outlines the benefits and facilitators from previous AIC and offers recommendations specific to digital health.

## Introduction

Digital health remains a growing and challenging niche in public health practice, particularly given the COVID-19 pandemic (1–5). Increasingly, the use of digital technology to deploy health programs are often thwarted by challenges of engagement, reach, sustainability, dissemination, evaluation, and equity (6, 7). Such digital health challenges persist in the business, technology, and health-care literature. Lagging research methods limit meaningful evidence, thus restricting understanding of efficacy and quality of digital health solutions (8). Digital health end-user engagement remains complex, demanding personalization and adaptive interventions that meet the needs for diverse populations (9). Health-care reimbursement complicates adoption and implementation, determining scale, and sustainability. Privacy and security issues raise warranted ethical discussions, thus demanding regulatory bodies to intervene with policy (10). Oftentimes, academic-industry collaboration (AIC) is endorsed as a mechanism to mitigate these barriers in digital health (11, 12). In response to the global COVID-19 pandemic, many digital health AIC emerged bringing cross-disciplinary sectors together (13–16).

Historically, diverse industries (e.g., semi-conductor, agriculture, space) leveraged AIC for scientific discovery and economic growth (17, 18). AIC has been observed in many clinical applications, yielding breakthroughs in biotechnology (biotech) and biomedicine (19, 20). More recently, in response to COVID-19, the company WHOOP partnered with universities and health system partners to clinical evaluate and deploy their digital respiratory wearable device (16). Similarly, companies like Apple and Fitbit collaborated with Duke University to monitor COVID-19 symptoms during sleep (21).

Many modern models of collaboration exist in digital health, reflecting traditional research and development (R&D) and innovative collaborative approaches [e.g., digital health ecosystems (22), competitions (23), cross-sector consortiums (24, 25), and industry partnerships with academic medical centers] (26–28). Despite AIC advocacy, hesitation remains due to the commonly reported misalignment between academia and industry. In the digital health field partnerships remain challenging due to conflicting priorities (29), values (30), approaches (31, 32), and expectations (33). Among ethical skepticism toward collaboration (34–37), barriers for collaboration include the initiation, maintenance, and sustainment of AICs (38–40).

The literature adequately reports challenges for AIC; however, it lacks a synthesis of benefits (e.g., outcomes) of partnerships and facilitators that guide such collaborations specific to digital health. With ongoing endorsement for AIC in digital health (11, 12, 27, 41, 42), initial investigation is needed to build consensus on best practices and approaches. The purpose of this mini review describes the benefits and facilitators of historical AIC and offers potential AIC recommendations specific to the digital health context.

## Methods of Mini Review

The mini review informed a larger, more robust scoping review on the topic (43). Using the PRISMA Extension for Scoping Reviews (PRISMA-ScR) (44) as a guide, the mini review formatively examined benefits, barriers, and facilitators of collaborations between academic and industry in digital health. During May 2019–December 2019, the researchers searched an online database (i.e., Ovid MEDLINE) to identify published papers that examined AIC in digital health settings. Eligibility criteria included both qualitative and quantitative studies, published or translated in the English language, and mention controlled keywords and MeSH subject headings. Example search terms included: digital health (e.g., mHealth, eHealth, innovation, technology, mobile applications, telemedicine, electronic mail, technology, text messaging, information technology, virtual reality); AIC (e.g., public or private, academic, University, industry, startup, cross-sector, collaboration, partnership, cooperation, linkage); recommendations, barriers, and/or facilitators. No date range was included in the eligibility criteria due to the formative nature of the mini review. Search results were exported using software application (i.e., Endnote) for screening and full-text review. Abstracted data included: benefits of collaboration, barriers, facilitators, and recommendations for collaborations. The abstracted data synthesized a range of evidence where sources were thematically grouped and analyzed based on extracted data.

## Benefits of Collaboration

Historically, AICs generate public health and economic benefits (i.e., outcomes of partnerships) (45). The literature reiterates that creativity and innovation emerge from heterogeneous perspectives (46, 47), and AICs serve as an intersection to advance that mission. (48) As economic drivers, AICs typically support businesses while leveraging University knowledge and insights (48, 49). Integrating academia and digital health industry improves translational medicine (50), particularly by creating business models that scale interventions to relevant populations. Such cross-sector alignment have reported helping health-care settings achieve the triple aim: (51) improving population health, care experiences, and reducing costs (52). As evidence accumulates, such comprehensive benefits of collaboration are projected to support the digital health field.

AIC cultivates innovation, benefiting both industry and academia. Both sectors may leverage partnership to maintain competitive advantage and advance science. The products from AIC allows industry to differentiate from competitors with scientific claims (39, 53). AIC better positions industry to profit when aligning with subject matter experts (54), improving regulatory appeals (55) and accessing innovative human capital (56, 57). Academic collaborators value from additional funding streams (39, 58, 59) and access to relevant innovations, inspiring new research directions (58). In addition, academic researchers benefit from the ability to extend evidence-based strategies to market for improved health outcomes (60), thus exploring commercialization as a sustainable form of dissemination (61). Such evidence suggests the outcomes of AIC are not unique to each respective field. Positive implications of AIC remain a standard product of interdisciplinary collaboration. Despite the reported benefits of AIC, initiating and executing partnerships in the real world proves challenging. The understanding of such partnerships remains limited (48, 62), particularly how these collaborations play out in the real world (63). While the literature adequately addresses barriers of collaboration, there lacks understanding of what facilitates digital health AIC, specifically the drivers that help guide the initiation and implementation of these partnerships in the real world.

## Academic-Industry Collaboration Facilitators

Many sources describe high-level lessons learned that facilitate partnerships among academic and industry collaborators. Such facilitators may be applied to digital health, particularly how they relate to interpersonal dynamics, context, and understanding of success. The importance of collaboration engagement relies heavily on the ability to establish and maintain authentic relationships with all stakeholders (64). Shared expectations, co-creation, awareness of diverse values, and flexibility are commonly described in the literature as facilitators to AIC (18, 48, 65–67). Collaborators should remain reflexive, anticipating, reframing, and negotiating interests (48, 65). Such interpersonal attributes reiterate the importance of trust (48, 68, 69) and interdependency (11, 69), adopting a shared mutually understood language to promote productivity (7). Most sources report establishing a reciprocally beneficial relationship rooted in transparency of each collaborators' values and motivations (52, 70). The acknowledgment and appreciation of interpersonal factors facilitate AIC (18, 20, 48, 52, 56, 68–72).

Beyond a general awareness of strengths and weaknesses facilitating AIC, collaboration climate remains a key facilitator for partnership. Models of collaboration vary; however, intentionally initiated (34, 48, 65, 73), co-created (66, 74–76), well-defined workflows enhance collaboration and improve the quality of deliverables. Many facilitators of AIC rely on a joint structure (66, 71) to leverage strengths of each discipline, encouraging flexibility when possible. (65, 77) Many authors underscore role clarity, communication, iterative project management style, and phase of project or partnership impacted the AIC (18, 32, 48, 52, 56, 64, 71–73, 78–81). A reoccurring cadence of communication promotes partnership, specifically by creating structure around coordination and collaboration management (75, 82, 83). Understanding unique strengths, institutional factors, available infrastructure support, capacity, organizational climate, and overall readiness are reported facilitators of AIC (11, 52). Any gaps in understanding could be addressed early (32) and continually to better manage expectations throughout the collaboration (33).

To fully establish shared expectations, AICs recommend alignment of outputs and deliverables as an early facilitator of collaboration. Many outputs for AIC exist, such as patents, intellectual property, data ownership, and publications. Academic and industry collaborators report prioritizing deliverables differently; thus, a facilitator of partnerships ensures each collaborator obtains an equitable value from collaboration or end goal (32, 65). Similarly, the value for the partnership may differ; therefore, aligning on adoption (52) objectives and scientific advancements (84) prove beneficial for AIC. Determining scientific, economic, and social impact was a reported challenge for AIC; however, some sources recommended using system thinking principles to determine socioecological levels worth investigating in the real world (32, 79, 85). Many sources recommended evaluating and documenting learnings from partnerships (65), particularly to better inform future AIC in practice (40). Establishing evaluation expectations (e.g., results and performance metrics) is important for goal alignment but also determining the success of collaboration. “Successful” AIC varies, but many sources report metrics around balance, mutual benefit, partnership sustainability, and co-development and defined dissemination strategy to facilitate collaboration (32, 52, 56, 72). Such shared expectations and acknowledgments of diverse goals facilitate AIC across disciplines and may offer promise in the digital health field.

## Recommendations for Digital Health Academic-Industry Collaboration

The mini review highlights the reported facilitators of AIC across many disciplines. First, on an interpersonal level, partnerships might consider initiating and implementing collaborations with trusted long-term relationships due to the historical differences between academia and industry. Leveraging trusted partners may improve shared values, clear roles and responsibilities, and encourage better team dynamics. Second, digital health AIC may consider thoughtful consideration of collaboration structure and knowledge sharing to promote understanding of business and market needs and scientific rigor. Given the nuances of technology and health interventions, project management that balances both design and research may promote flexibility, speed, and science. Third, while measuring success proves challenging, digital health AIC may consider setting clear expectations on the dissemination, implementation, partnership, and performance of the digital health product to avoid common conflict around evaluation and ownership.

Due to the barriers reported in the digital health field, such facilitators serve as an initial roadmap to guide both researchers and digital health companies looking to advance their technologies. Currently, there lacks consensus on models of collaboration relevant to digital health (18, 48, 86). Table 1 offers practical solutions, in the form of a check list for initiating and maintaining AIC in the digital health field.

**Table 1:** Checklist of Recommendations for Digital Health AIC.

**Interpersonal Recommendations**
**Foster long-term relationships aid in collaboration initiation and implementation**
□ Consider leveraging personal and/or professional network to initiate AIC □ Avoid transactional partnerships (e.g., one-off projects or contracts) or last-minute urgently requested collaborations □ Consider community-based participatory research best practices when convening all collaborators □ Practice health literacy principles and remain culturally responsive to each sectors' terminology □ Allow strengths of each collaborator to thrive (e.g., science, research methods, product development, market research, commercialization) □ Verbally identify and agree on areas of complementary strengths to build mutual respect
**Build trust among collaborators**
□ Allow time for trust to establish among collaborators, avoiding transactional partnerships □ Assume good intent for all parties involved, anticipating differences □ While convenience partnerships are ideal for trust, diversify AIC with new collaborators to foster innovation and avoid groupthink □ Raise and address health equity considerations for digital health collaboration
**Level set on shared value of collaboration**
□ During ideation phase, raise awareness of values and co-create action plan to complement diverse values □ Establish buy-in from all stakeholders to encourage characteristics for success and alignment of goals/deliverables □ Outline values and motivations contractually and verbally during initial collaboration stages □ Conduct value proposition exercises to bring clarity on diverse interests early in collaboration □ Judiciously negotiate conflicting interests to mutually benefit from partnership
**Implement clear roles and responsibilities**
□ Define role clarity and areas of dependence of other partners □ Hire talent with experience in multiple industries, translating between sector's terminology □ Ensure industry partner obtains a doctoral-level degree, serving as the scientific “broker” between sectors □ Convene early to openly discuss diverse value propositions □ Encourage regular cadence of meetings (appropriate for phase of project/product) to connect on issues pre-emptively
**Encourage positive and product team dynamics**
□ Promote inclusion and diversity best practices to establish an inclusive culture □ Resolve conflicts throughout collaboration rather than waiting for project to finalize □ Execute principles of teamwork with collaborators as you would your own internal organization
**Contextual recommendations**
**Initiate collaborations thoughtfully**
□ Identify a “formula for best collaborator fit” based on situation (e.g., phase of project, type of product, collaborator attributes) □ Recognize and address sources of conflict early during collaboration initiation □ Identify and discuss institutional or organizational structures that may limit flexibility (e.g., IRB timelines, legal processes, indirect costs) □ Establish formalized agreements, confidentiality agreements, and encourage intellectual property discussions □ Define scope of work and delegate responsibilities to maximize strengths and expertise
**Define a collaboration structure (e.g., governance, partnership model)**
□ Consider a governance structure or larger stakeholder body guiding the work □ Capture diverse subject matter expertise and end-user input for all projects/collaborations □ Ensure all stakeholders are a part of the collaboration structure, including non-profits, community partners, and government bodies □ Create opportunity for end-user (e.g., patient, consumer) to engage in structure □ Agree on coordination preconditions for collaborative decision-making and partnership implementation
**Promote knowledge sharing across collaborators**
□ Each sector has a responsibility to inform other collaborative party of knowledge gaps (e.g., IRB, legal, human subjects, subject matter expertise, UX/UI best practices) □ Collaborators are not expected to be experts but aware of processes (e.g., IRB or FDA approval, academic budget restraints) □ Create a knowledge sharing environment and avoid penalizing other sector for not obtaining expertise □ Create social and physical environments that facilitate cross-sector learning (e.g., workshops, kickoff meetings, lunch, and learns) □ Create a culture of collaborative learning for collective impact, including end-users, community stakeholders, collaboration key players at all levels of organizations
**Execute rigorous project management**
□ Hire project/product managers on both sides of collaboration to execute the day-to-day work □ Identify collaboration champions to serve as decision-makers and brokers between parties □ Create a list of anticipated blockers that may inhibit timeline and strategize methods to overcome □ Share forecasted or upcoming barriers during meetings, virtually, or in-person □ Meet often during initial stages and regularly during AIC to avoid challenges that arise
**Blend both design and research for digital health AIC**
□ During the design, consider how products/services many intervene on individuals and communities (social ecological model) □ Build business cases (e.g., regulatory guidelines, commercialization) to offer sustainable dissemination strategies □ Advocate and instruct on scientific best practices (e.g., IRB, outcomes research, human subjects) to promote scientific rigor and attention to health inequities □ Discuss unintended consequences of digital health product or project early to avoid pitfalls □ Create logic models and discuss societal and environmental impacts during design and development □ Industry should differentiate between the execution of product research (UX/UI) and outcomes research (clinical trials)
**Value an iterative human-centered design approach and principles**
□ Train partners in human-centered design phases (i.e., empathy, define, ideate, prototype, test) □ Allow for iterative approach both in research and product development □ Include trainings and iterative approaches into grants and contracts for flexibility and accountability
**Expectation recommendations**
**Set expectations about collaboration implementation**
□ Create expectations based on both academic and industry interests and timelines, document contractually and discuss orally □ Align objectives with defined contractual agreements (e.g., data ownership, legal rights) □ Understand costs for clinical validation and fees for service (e.g., indirect costs, full-time equivalent salary costs, publication fees)
**Create metrics of success and evaluate collaboration progress**
□ Similar to the scientific process, evaluate short- and long-term outcomes of AIC and the digital health product □ Consider capturing relationship dynamics through surveys, interviews, or working group meetings □ Conduct retrospective meetings to discuss what worked, what didn't work, and areas of opportunity with each sector's stakeholders □ To avoid loss of knowledge or partnership understanding; document partnership as much as a product portfolio or grant deliverable □ Track process and results for quality improvement throughout collaboration
**Evaluate the design and the performance of the digital health product**
□ Consider identifying academic collaboration in the design of intervention to serve as subject-matter experts □ Initiate collaboration with already commercialized products to build evidence □ Recommend iterative clinical validation of digital health products (e.g., feasibility, usability, clinical trial) prior to full-scale commercialization □ Avoid consumer-level digital health products (e.g., B2C), as it only impacts individual *via* Public Health Pyramid □ Improve evaluation methods using innovative models of analysis □ Capture end-user feedback (e.g., patients), system professionals (e.g., providers), and system leadership (e.g., hospital administration)
**Establish shared expectations for dissemination**
□ Outline methods of reach and scale for broad adoption, building on business models specific to digital health interventions □ Consider sustainable methods found in the business model literature (e.g., B2B or B2B2C) □ Work with tech transfer offices or legal to determine options for intellectual property and data handling □ Early in AIC, set up publication agreements and discuss intellectual property early in negotiation and contracting □ Publish case studies describing experiences with AIC and digital health product outcomes

## Discussion

This mini review offers valuable insight for the wider scientific and digital health community, underscoring the opportunity in collaboration for public health purposes, serving as a blueprint for digital health researchers seeking industry collaboration. As the use of digital health continues to grow, it challenges practitioners, researchers, and technologists to explore ways to advance technologies for health-care delivery, disease prevention, and public health. Public-health efforts such as education, policy, research, and service implementation remain critical for advancing public health initiatives, however underscore the importance of collaborative practices (87). The collaboration literature echoes this philosophy, illustrating the benefits of heterogenous perspectives when solving for complex situations (47). Stakeholder engagement remains a responsibility of public health, engaging government, nonprofit organizations, and diverse community partners. Industry, particularly the digital health industry, is an additional key stakeholder for disease prevention and health promotion efforts.

In the last few years, researchers and policy makers endorsed interdisciplinary collaboration and future efforts to advance digital health technologies (11, 88, 89). This cross-sector collaboration sentiment is shared by top global health officials in response to the COVID-19 pandemic. The World Health Organization (WHO) presented a call to action for “a global collaboration to accelerate the development, production, and equitable access to new COVID-19 health technologies.” (15) Various innovative diagnostics, therapeutics, and digital tools offer promise in mitigating the social and economic consequences of the COVID-19. Global WHO leaders recognize the alignment, urging community stakeholders and political leaders to commit to a shared aim of equitable global access to innovative tools for COVID-19 for all, emphasizing collective problem-solving, establishing mutual benefit where all stakeholders can share their expertise and knowledge in a productive and meaningful way (15). With such advocacy, AIC remains an emerging frontier to navigate in the digital health industry for both academia and industry.

While the mini review obtains limitations, it offers initial snapshot and recommendations based on historical success of AIC in other industries. The work warrants a scoping or systematic review to fully understand the scope of the collaborative practices in digital health. Findings of the mini review informed a more robust literature search on the topic with clearly defined boundaries and search criteria. The findings of this mini review offer actionable collaboration insights for academia and industry to respond to this call to action. Alignment and partnership with diverse stakeholders prove challenging, however, historically demonstrates rewarding. Many high-functioning AICs have been operating for years and evolved together over time. Processes and extent of collaboration varies; however, acknowledging and appreciating differences may bolster these partnerships and shorten the learning curve among collaborators.

WHO's call to action reiterates the importance of AIC and balancing strengths to create global change by both advancing science and supporting the economy. It remains insufficient for research produce evidence and fails at scale and implementation. Commercial products on the market must be evidence-based and obtain real-world value. Academia and industry are operating in a highly complex adaptive system, where its critical to work together to translate evidence in the real world at scale. Digital health AIC may be an important synergy to answer WHO's call to action.

Despite the growing interest in digital health as a solution for public health problems, challenges persist. While AIC is continually endorsed to alleviate some of these challenges, limited knowledge is known on partnership best practices specific to digital health. To fully realize the potential the digital health industry obtains, stakeholders must be willing to recognize and leverage the strengths of each discipline to drive the industry and public health forward.

## Author Contributions

Under the supervision of SB, SM, and KF designed the review and conducted the investigation as a part of a larger body of research. KF and JP developed and synthesized recommendations. KF initiated the manuscript with JP. SZ, XZ, and SG contributed iterations and manuscript edits for final submission. All authors agree and are accountable for the content of the work.

## Conflict of Interest

The authors declare that the research was conducted in the absence of any commercial or financial relationships that could be construed as a potential conflict of interest.
